# Oral Health Promotion in Pediatric Age Groups: Habits and Behaviors of Italian and Spanish Parents and Children

**DOI:** 10.3390/jcm14061926

**Published:** 2025-03-13

**Authors:** Francesco Mariotti, Giulia Zumbo, Francesca Ripari, Jorūnė Emilija Valaikaitė, Matteo Mariotti, Antonella Polimeni, Iole Vozza

**Affiliations:** 1 Department of Oral and Maxillo-Facial Sciences, Sapienza University of Rome, 00161 Rome, Italy; mariotti.1734079@studenti.uniroma1.it (F.M.); francesca.ripari@uniroma1.it (F.R.); antonella.polimeni@uniroma1.it (A.P.); iole.vozza@uniroma1.it (I.V.); 2 Faculty of Medicine, Vilnius University, M. K. Čiurlionio Str. 21, 01513 Vilnius, Lithuania; jorune.valaikaite@gmail.com; 3 Department of Economics, Universidad Complutense de Madrid, 28040 Madrid, Spain; mmariott@ucm.es

**Keywords:** oral health, oral prevention, oral promotion, dental caries, oral health habits, pediatric dentistry, parents, children

## Abstract

**Objectives**: The aim of this study was to evaluate the level of knowledge and awareness among parents regarding oral hygiene habits in two different national groups and to raise awareness among parents about oral hygiene education. **Methods**: A sample of 640 parents from Rome (Italy) and Valencia (Spain) was collected. The survey involved children between 0 and 18 years of age. An online questionnaire was administered to gather information regarding demographic data, the level of knowledge about dental caries and its transmission, proper oral hygiene habits of parents with regard to their children, mothers’ attitudes towards their own oral health before or during pregnancy, awareness of risk behaviors, such as the use of pacifiers or baby bottles, sharing cutlery, the use of the same toothbrush in the entire family, the role of schools, and oral health prevention techniques. **Results**: The comparison between the two groups shows that Spanish parents are more attentive to oral hygiene measures compared to Italian parents, especially about the knowledge of dental caries and its transmission, oral health check-ups during pregnancy, and proper oral hygiene habits. In both groups, there is still little knowledge about oral prevention methods. **Conclusions**: From the results of our survey, we can conclude that the knowledge regarding oral hygiene among parents from both countries is not yet optimal when compared to international health objectives. It is necessary to promote oral health prevention programs both in schools and within families in order to improve children’s oral health.

## 1. Introduction

Despite significant progress in general and oral health, oral pathologies, particularly dental caries, continue to be highly prevalent in pediatric age groups [1]. Kassabaum et al. [2] demonstrated through a systematic review that over 2.4 billion people suffer from dental caries, with more than 621 million of them being children. The consequences of dental caries in children are evident; in addition to orofacial pain, they may experience sleep disorders [3], unhealthy dietary habits [4], speech and growth disorders, and weight loss [5]. Moreover, the development of dental caries in deciduous dentition increases the likelihood of caries in permanent dentition. All of these factors can impact a child’s performance and academic efficiency [6]. As a multifactorial condition, dental caries is known to be associated with cariogenic foods, as well as poor oral hygiene habits. The WHO [7] emphasizes that knowledge of dental caries risk factors can decrease its development, with the aim of developing prevention strategies and promoting oral health. Schools should actively participate in these community strategies as children spend most of their time there. A healthy school environment leads children to adopt a proper lifestyle that they will maintain throughout their lives [8]. Schools profoundly influence children’s well-being and development [9]. Schoolteachers are considered role models for transmitting correct life values and play a central role in establishing habits that promote health from an early age. They contribute to shaping behaviors, ideas, and attitudes related to health [10]. Therefore, it is essential to increase teachers’ knowledge about general and oral health to impart these values to children [11,12]. Bramantoro et al. [11] also report that school oral hygiene programs are highly effective, yielding positive outcomes in terms of behaviors, knowledge, and quality of life. Despite a slight reduction in dental caries pathology in recent years through the implementation of prevention strategies directed at families, the role of schools has been largely neglected. There is still a lack of health education within schools.

The role of the pediatric dentist in preventing the early development of such lesions is key to improving children’s oral health, along with the pediatrician’s influence on a child’s awareness of systemic health importance. Unfortunately, low socioeconomic status affects the level of healthcare, along with income, education level, and family size [13,14,15]. Prevention and early intervention are crucial, especially in pediatric patients. Parents serve as role models for their children, teaching them correct lifestyles that promote well-being and good health. Children, in fact, do not have autonomous awareness of their own health, and this concept needs to be conveyed by parents.

The aim of this study is to evaluate the level of knowledge and awareness among parents regarding oral hygiene habits in two different national groups and to raise awareness among parents about oral hygiene education.

## 2. Materials and Methods

This study was carried out by administering an anonymous online questionnaire using the Google Forms platform. Data were collected between 1 August 2023 and 20 October 2023 in the cities of Rome and Valencia. The subjects were chosen randomly from a range of volunteers and patients who visited hospital clinics in Rome and Valencia during the mentioned time period. A random sampling technique was used in this study.

A QR code was displayed to the patients who went to the clinic, while a link with the questionnaire was provided to the volunteers. The link was shared on major communication platforms such as WhatsApp and Instagram. The questionnaire’s validation was previously confirmed in a study by Vozza et al. [16]. It was expanded and approved by the ethics committee of Sapienza University of Rome (Protocol n.3101/23), according to the previous questionnaire conducted by Vozza et al.

The questionnaire was administered to 291 Italian parents and 349 Spanish parents with children aged between 0 and 18 years. The questionnaire was titled “Experimental Study: Oral Hygiene Habits, Parents, and Children” and it was translated into Italian and Spanish.

The study protocol adheres to the Helsinki Declaration guidelines from 1975 and was approved by the ethics committee of Sapienza University of Rome (Protocol n.3101/23).

The inclusion and exclusion criteria for this study were as follows.

Inclusion Criteria:

- Parents of children aged 0–18 years living in Rome (Italy) or Valencia (Spain).
- Parents who provided informed consent to participate in the study.
- Participants who completed the questionnaire in its entirety.

Exclusion Criteria:

- Parents who did not provide informed consent.
- Incomplete questionnaire responses.
- Parents not from the designated study locations (Rome and Valencia).
- Parents of children with medical conditions or syndromes that could significantly impact oral health, such as genetic disorders affecting dentition.

The parents of the children were informed about the purpose of the experimental study, and informed consent was obtained before answering the questionnaire. The questionnaire opened with the message: “*Dear Parents, the following questionnaire is part of a research study concerning the awareness, knowledge, and importance of pediatric oral hygiene habits among parents, particularly aimed at preventing dental and maxillofacial alterations. Thank you for your cooperation; it will not take more than 5 min. This study is conducted by a research team from Sapienza University of Rome, and we will treat all responses anonymously, analyzing the results for statistical purposes only. Participation in the questionnaire implies consent to the processing of entered data*”.

For both populations, the same questionnaire was administered, with identical questions and multiple-choice answers, with the language adjusted according to the country of origin to maintain the statistical validity of the survey. The questionnaire was divided into 7 sections. The total number of questions is 29 with multiple-choice answers in both Italian and Spanish. Table 1 shows the aforementioned structure.

The results were collected in a Microsoft Excel 10 database (Version 16.32, Redmond, WA, USA, 2023), and descriptive statistics were developed for each element using tables and comparison graphs between the two different samples.

## 3. Results

A total of 640 parents with children aged between 0 and 18 were involved in this study, 291 of Italian nationality and 349 of Spanish nationality. Section I of the questionnaire reports the data regarding parents and children. Among Italian parents, 46% claimed it was their first experience, while 54% stated that they had more than one child. On the other hand, in the Spanish sample, 71.1% stated that it was their first experience, leaving the remainder with prior experience. The final analysis involved 291 Italian and 349 Spanish children. Table 2 shows the children’s age for both groups. It can be noted that the distribution of the Italian sample in the city of Rome and its province, with the questionnaire identifying 132 girls and 159 boys, is biased toward children aged between 9 and 12 years. In contrast, in the Spanish sample, including children in the city of Valencia and its province, responses were collected from 162 girls and 187 boys, most of whom were aged between 0 and 3, representing the majority of the responses collected. Out of the 291 Italian parents, 240 were mothers, and 51 were fathers; among the 349 Spanish parents, 333 were mothers and 16 were fathers. Table 3 illustrates the percentage of children who suffer some kind of systemic pathology, with oral pathologies excluded. Results of others previously mentioned sections are reported and summarized in Table 4, Table 5 and Table 6. Clearly, the majority of children in both samples were healthy.

### 3.1. Italian Questionnaire Analysis 

#### 3.1.1. Section II: Knowledge of Dental Caries

**Figure 1:**
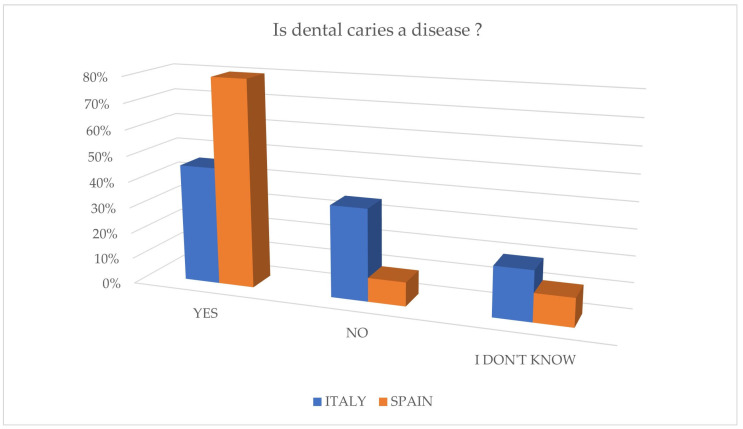
Level of knowledge of dental caries as a disease by both groups in the study.

In this section, only 45.4% of the 291 Italian parents recognized dental caries as a pathology. Moreover, 75.6% believed that it was not transmissible, and 12.4% responded positively. Approximately 46.7% of children experienced dental caries, with 93.4% of them receiving treatment, while a small percentage left the carious disease untreated. Despite a lack of information about caries origin, 81.1% of parents agreed that decayed milk teeth should be treated (Figure 1—Section 4: Discussion).

#### 3.1.2. Section III: Parental and Child Behavioral Habits

Despite the low awareness of dental caries, data regarding positive oral hygiene in children habits emerged. Notably, each family member owned a toothbrush, and 90.4% did not share utensils during meals. About 55% breastfed their child, while 22.7% and 22.3% used bottle and mixed feeding, respectively. The prevalent duration of breastfeeding was between 6 months and 1 year, accounting for 48.5%, while 25.8% breastfed for less than 6 months and 25.8% breastfed for more than one year. In addition, 37.8% did not use any device, while others used pacifiers (34.4%) and milk in bottles (26.8%). Most parents indicated that their child used devices for reasons like sleep or to soothe crying.

#### 3.1.3. Section IV: Pregnancy

Among the 240 surveyed Italian mothers, 64.4% visited a dentist during pregnancy for various reasons, especially for a check-up (39.4%) and oral hygiene (38.1%). In addition, 11.6% stated that it was for dental caries, while 11% stated that it was for orofacial pain. Unfortunately, only 12% of mothers were advised by their gynecologist to attend an oral screening, while 61.7% received no such recommendation; moreover, 25% stated that it was as a result of their own initiative, while 1% were advised by other specialists.

#### 3.1.4. Section V: Knowledge of Home Oral Hygiene

The results showed that 52.6% started brushing their child’s teeth between 2 and 3 years, while 76.3% used age-appropriate toothpaste. However, 17.9% used the same toothpaste for everyone, and 7.9% used toothpaste with hard bristles.

#### 3.1.5. Section VI: The Role of the School

Concerning the school’s role, 41.2% of Italian children had snacks like fruit juice or candy bars during the school break; on the other hand, only 11.3% of the children had the opportunity to brush their teeth after lunch. Effectively, 75.7% indicated no such opportunity at school.

#### 3.1.6. Section VII: Prevention

Regarding dental preventive care, only 40.5% of Italian parents were aware of dental sealants, and most (70.4%) had not administered fluoride drops or tablets to their children in the first three years.

### 3.2. Spanish Questionnaire Analysis

#### 3.2.1. Section II: Knowledge of Dental Caries

In this section, a significant 79.9% of the 349 Spanish parents correctly recognized dental caries as a disease. However, like the Italian respondents, when asked about the transmissibility of dental caries vertically or horizontally, only 25.5% of the sample responded accurately (Figure 1—Section 4: Discussion). Nevertheless, only a small percentage of children had experienced caries (13.8%), out of which 93.75% received treatment. Impressively, 95.1% of parents were aware of the necessity to treat decayed primary teeth.

#### 3.2.2. Section III: Parental and Child Behavioral Habits

Concerning habits, no parent shared a toothbrush with their child, and a quarter of parents (25.8%) shared utensils during meals. Additionally, breastfeeding was prevalent (65.6%), followed by mixed (20.3%) or artificial feeding (14%). The duration of breastfeeding was mostly over 1 year, while 24.9% stated it was between 6 months and 1 year, and the remaining portion (18.1%) stated it was shorter than 6 months. Similarly, the use of devices like pacifiers or bottles with milk was reported, with the majority using them to make the child sleep (54.7%), to make them stop crying (22.4%), or for other reasons (22.9%).

#### 3.2.3. Section IV: Pregnancy

A high percentage (86%) of Spanish mothers attended a dental visit before or during pregnancy, mainly for check-ups (62.5%) or oral hygiene (29.5%). Yet, only 24.6% received advice from their gynecologist to have a dental check-up during the first trimester, while 1.4% received no such recommendation; moreover, 24.1% went based on their own initiative and 9.9% received advice from other specialists.

#### 3.2.4. Section V: Knowledge of Home Oral Hygiene

Regarding the practices related to oral hygiene among Spanish respondents, a significant majority (84%) began brushing their child’s teeth soon after the eruption of the first tooth. Moreover, 93.4% used age-appropriate toothpaste, and 96.3% used a toothbrush with a small head and medium or soft bristles. The importance of tongue hygiene was recognized by 95.7% of respondents.

#### 3.2.5. Section VI: The Role of the School

Regarding the school environment, during mid-morning breaks, Spanish children consumed various foods, predominantly ’Other’ items (52.7%) followed by sandwiches (36.1%). A significant proportion (66.2%) of children had lunch at school, but, unfortunately, only 25.5% of schools allowed students to brush their teeth after lunch, with 70.1% lacking such provisions.

#### 3.2.6. Section VII: Prevention

In terms of preventive measures, only 37.5% of Spanish parents were aware of dental sealants, while a substantial majority (62.5%) had never heard of them. Additionally, 88.3% did not administer fluoride drops or tablets to their children during the first three years of life.

## 4. Discussion

The results show significant differences between the two groups.

Four main pillars emerge as critical points that require intervention: the transmission of dental caries from mother to child, harmful oral habits such as the use of pacifiers and bottles with sugary drinks, home oral hygiene, and the role of schools in promoting oral health.

**Figure 2:**
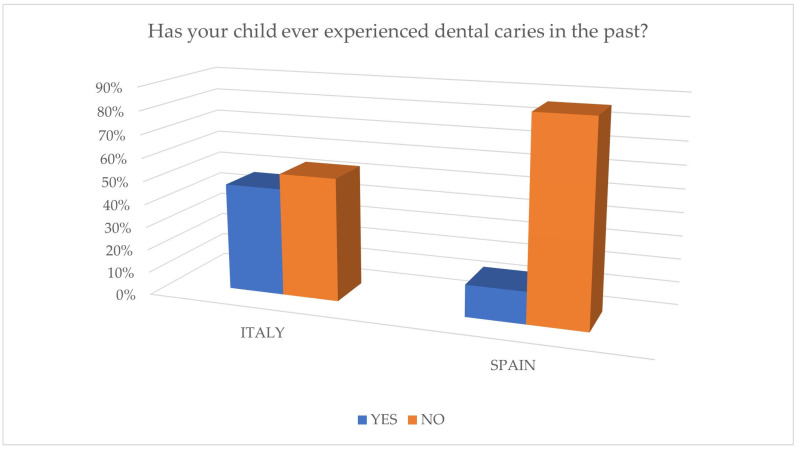
Percentage of children who have experienced dental caries in the past.

The data collected from the questionnaires highlight differences in the knowledge of caries between Italian and Spanish parents, with greater attention to caries prevention by Spanish parents, as shown in Figure 1 and Figure 2. Italian children show a higher previous experience of caries (46.7%) compared to Spanish children (13.8%), indicating greater awareness and attention to prevention in the Spanish population. These findings are consistent with official reports for the involved countries, as in Italy, the percentage of experience of tooth decay in children is higher when compared to the official data reported by the Italian Ministry of Health [17]; in Spain, it is notably lower when compared to the data from the Oral Health Survey 2020 [18]. The treatment of caries in children with previous experience has had positive outcomes in both populations.

**Figure 3:**
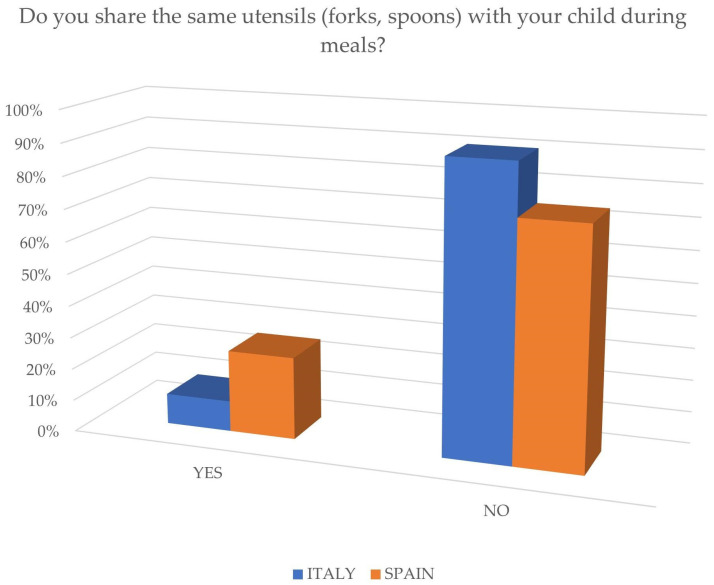
Sharing utensils during meals.

Regarding oral hygiene, both groups use different toothbrushes for different family members, but there were differences in the use of utensils during meals, with a higher percentage of 25% in the Spanish group compared to less than 10% in the Italian group, as shown in Figure 3.

Breastfeeding was prevalent in both groups, with a duration of less than 1 year for the Italian sample and over 1 year for the Spanish one. The WHO [7] recognizes breastfeeding as the ideal option for children during the first 6 months. In fact, the beneficial effects of breastfeeding are known for both the child [19] and the mother [20].

**Figure 4:**
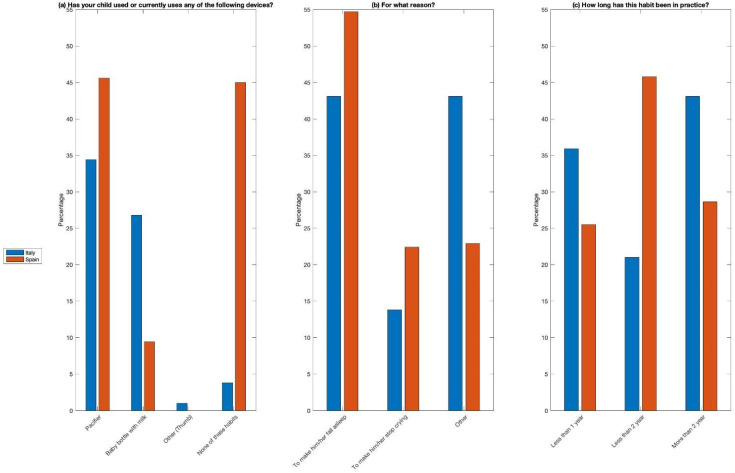
Different behavioral habits of Italian and Spanish children: (**a**) use in the past or current use of some devices such as a pacifiers, baby bottles with milk, other (thumb), or none of these habits; (**b**) reasons to use these devices; and (**c**) time using these devices.

The use of pacifiers did not show significant differences between the two samples, while the use of bottles with milk was more prevalent among Italian parents (27%), compared to nearly 10% of Spanish parents. The duration of use of such devices extended beyond 2 years in children of Italian nationality, while it was under 2 years for those of Spanish nationality. Figure 4 reports these results.

**Figure 5:**
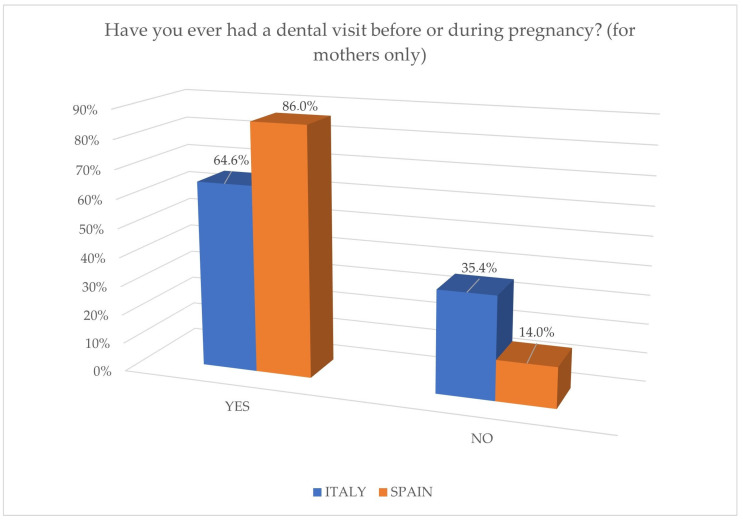
Behavior of mothers before or during pregnancy.

Regarding the role of pregnancy, the need to include a dedicated section has been assessed, considering the impact of hormonal changes on the onset of oral conditions [21], such as periodontal disease [22] and gingivitis [23]. However, from the results, we can affirm that the majority of mothers from both groups adopted proper behavior by visiting the dentist during pregnancy for a check-up or professional hygiene, as reported in Figure 5. This demonstrates awareness regarding the importance of maintaining proper oral hygiene during pregnancy, with the aim of enhancing the quality of life of the mothers themselves [24].

**Figure 6:**
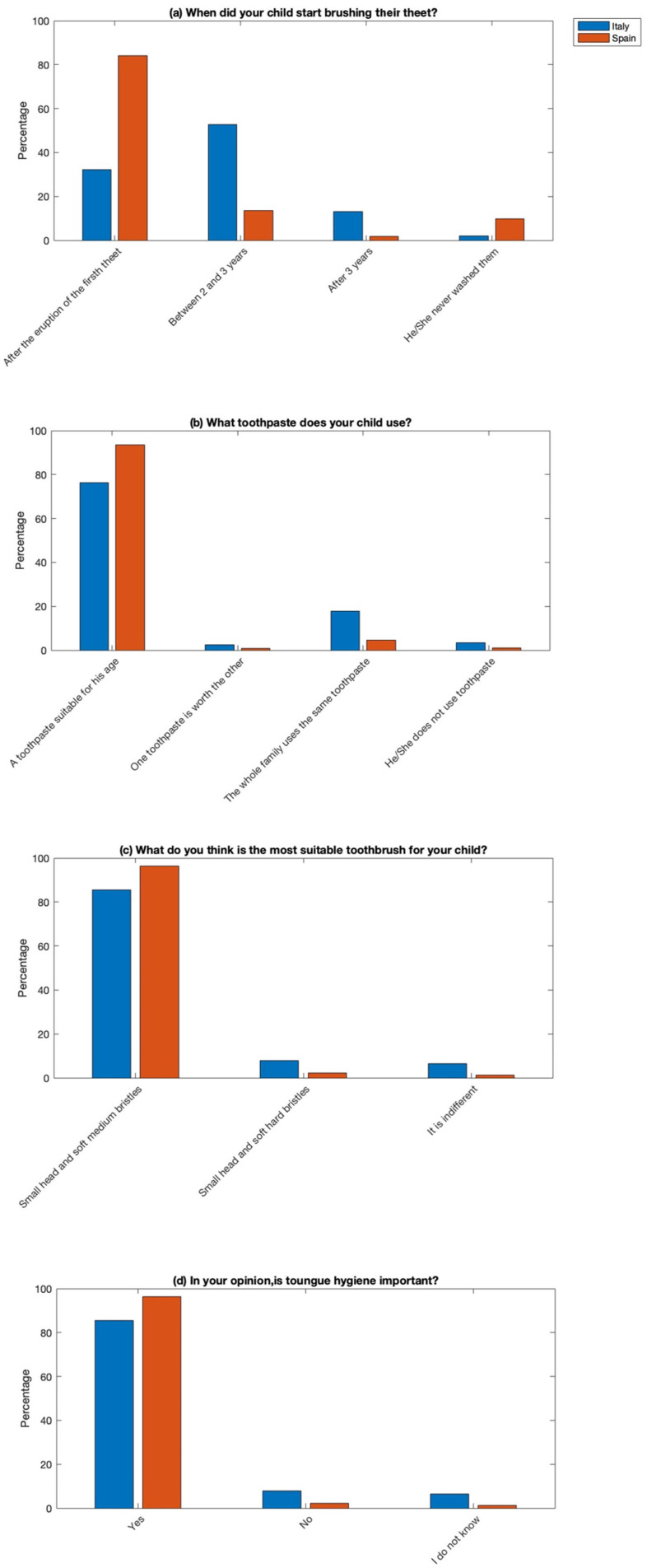
Parents’ knowledge about home oral hygiene: (**a**) start of toothbrushing of the child; (**b**) type of toothpaste appropriate to the child’s age; (**c**) type of the most suitable toothbrush; and (**d**) the importance of tongue hygiene.

About the section concerning the level of knowledge about home oral hygiene, the data are quite consistent between the two populations examined regarding the use of toothbrushes, toothpaste, and tongue hygiene. However, there is slight divergence concerning the initiation of home hygiene. Figure 6 shows differences in the onset of oral hygiene for children, with a higher percentage of Spanish parents initiating brushing immediately after the eruption of the first temporal tooth, unlike Italian parents who tend to start later, between 2 and 3 years of age. This indicates a concerning trend for the Italian group, considering that the literature emphasizes the importance of initiating this procedure between 6 months and the first year of life [25].

**Figure 7:**
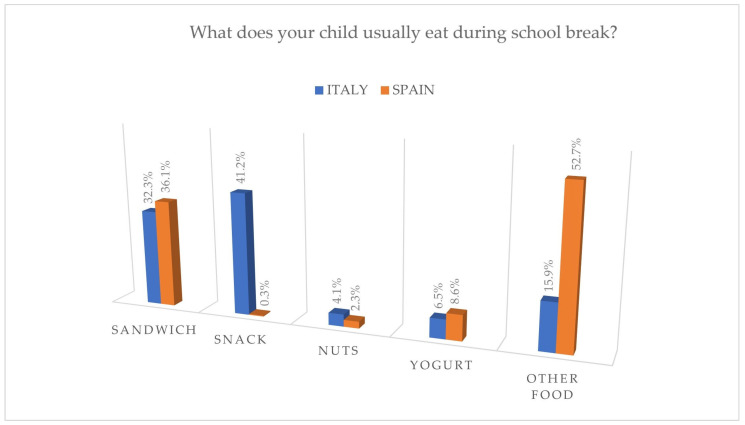
Food preferred by Italian and Spanish children during the school break.

The school appears to play a limited role in oral hygiene, with the majority of Italian children consuming packaged snacks and fruit juices during school breaks, while Spanish children prefer other foods, as shown in Figure 7. Furthermore, only a minority of schools encourage and give the opportunity to children to brush their teeth after lunch. It is true that this is not mandatory, but still, schools can play a significant role in the promotion of oral health, and therefore, prevention programs have been developed and are still active, aimed at intercepting and preventing oral cavity diseases from early childhood, recognizing schools as promoters of both general and oral health [8].

**Figure 8:**
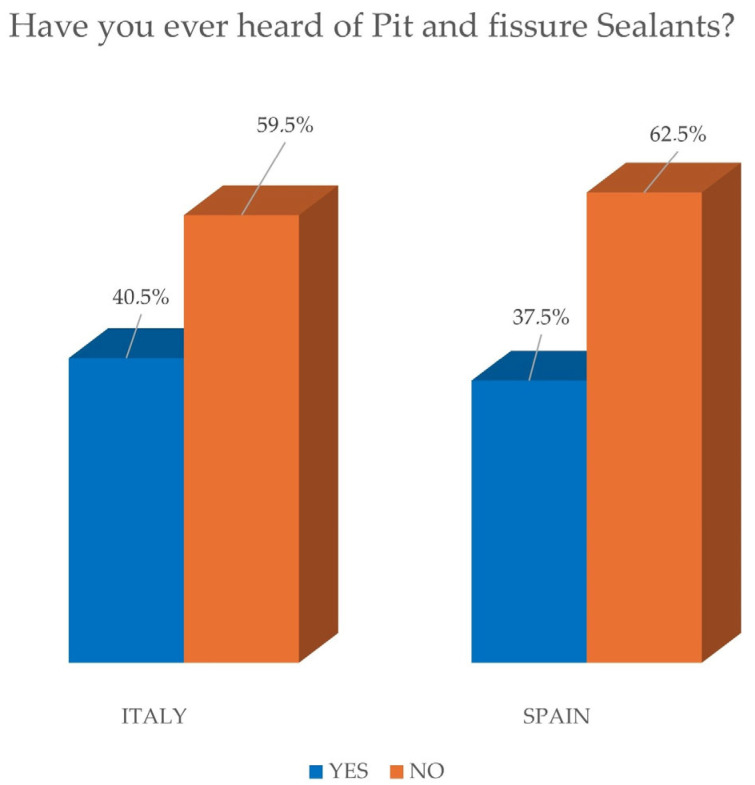
Knowledge of pit and fissure sealants.

Lastly, Figure 8 shows how there is little knowledge and adoption of preventive measures such as sealants and fluoride administration through toothpaste, indicating the need for greater information and promotion of these preventive practices. Sealants, in particular, have been recognized as a highly effective preventive technique for several years [26,27].

### 4.1. Transmission of Tooth Decay

This study highlights the significant involvement of mothers, focusing on the transfer of pathogenic oral bacteria to their children. The mother, being in close contact with the child from the beginning, plays a key role in the transmission of harmful bacterial flora that can affect the child’s oral health.

Sharing utensils during meals, tasting food or bottles, and the transmission of saliva are critical factors that favor the horizontal transmission of bacteria responsible for tooth decay [28]. This transmission can begin at the placental level [29,30] and can increase through incorrect daily behaviors of parents, especially mothers [31,32,33,34]. Therefore, during pregnancy and breastfeeding, it is important to ensure a healthy mouth [35,36].

Once transmitted, bacteria can multiply and thrive, especially if fermentable foods or sugary drinks are introduced through devices such as pacifiers or bottles during the night when saliva production is reduced.

Several studies demonstrate the vertical transmission of bacteria from mother to child [37,38,39,40,41,42] and that some strains of bacteria, such as S. Mutans, can be shared within the same family, as reported in Childers et al.’s study [43]. This confirms the potential horizontal transmission of bacteria [38,40,44,45]. The colonization of the oral cavity by S. Mutans already begins in post-natal children [33,46].

However, there are various individual variables that influence the child’s susceptibility to bacterial transmission, including factors such as the period of infectivity [47], the number of erupted teeth [48], the presence of enamel hypoplasia [49], sugar consumption [50], and the child’s immune system conditions [51].

The main goal of oral health professionals is to educate and instruct parents, especially mothers, on proper oral hygiene practices to reduce the presence of bacteria responsible for tooth decay and prevent the onset of the condition [52].

According to the results obtained, although the level of knowledge regarding the transmission of tooth decay is slightly higher among Spanish parents compared to Italian ones, the majority of parents in both groups are not aware of the possibility of transmitting pathogenic oral bacteria to their child.

### 4.2. Children’s Bad Habits

The results highlight the improper use of pacifiers and bottles, which can have negative effects on the dental and craniofacial health of children. Often, children between 1 and 5 years old develop incorrect habits such as pacifier sucking, finger sucking, or bottle feeding, behaviors that can cause dento-skeletal problems. The collected data show the prolonged use of these devices in both Italian and Spanish children, with a significant percentage of children using them beyond the age of two.

The prolonged use of pacifiers and bottles is associated with a high risk of developing Early Childhood Caries (ECC) [53], which clinically affects the vestibular surface of the teeth in the anterior region of deciduous dentition, quickly spreading to the rest of the dentition [54,55,56]. The literature also reports that the use of bottles and pacifiers beyond the early years of life poses a major risk for the development of irregular dentition in children [57], negatively influencing the alignment of permanent teeth. However, breastfeeding seems to be associated with a reduction in the incidence of malocclusion problems and promotes the proper development of the palate and oral muscles [20].

During sucking on the bottle nipple or pacifier, there is a greater likelihood of developing atypical swallowing patterns [58]. Prolonged pacifier use beyond 3 years can lead to problems such as anterior open bite, posterior crossbite, and a narrow palate [59].

In summary, the habit of using pacifiers and bottles beyond a certain age limit can have serious consequences that may even manifest in adulthood. It is crucial to inform parents about the negative effects these habits can have on the proper development of their children.

### 4.3. Home Oral Hygiene of Children

The American Academy of Pediatric Dentistry (AAPD) [60] recommends the proper brushing of teeth twice a day using a toothbrush with soft or medium-sized bristles that are suitable for children. Young children are not capable of brushing their teeth on their own, so it is the responsibility of parents to take care of their oral hygiene [61]. Gradual teaching of oral hygiene from a very young age helps the child learn and adopt these habits for a lifetime. Parents’ guidance in monitoring and guiding children in proper tooth brushing is crucial to reduce the risk of developing caries [62].

The study results indicate that the majority of Italian parents started brushing their children’s teeth late (between 2 and 3 years), with only 32% starting after the appearance of the first tooth. These data highlight a lower level of awareness among Italian parents compared to Spanish ones regarding managing their children’s oral hygiene. The observed data in the Italian population reflect the lack of comprehensive information and training among parents regarding the management of their children’s oral hygiene, a point confirmed by previous studies [63]. Early involvement of a dentist and the use of oral hygiene tools are essential to solidify the habit of oral hygiene and contribute to the reduction in oral diseases.

### 4.4. The Role of the School

The school environment plays a significant role in educating and promoting oral hygiene in children, especially considering the time children spend in schools. Teachers, as consistent figures in children’s lives, can become important sources of information on oral hygiene; therefore, the introduction of educational programs in this regard should be early.

Vozza et al. [64] in a study conducted on 70 children at an elementary school in Gaeta (Latina, Italy) found that although parents are attentive to their children’s oral health, periodic check-up visits are often insufficient to raise awareness and motivate children. The same results are also reported in Guerra et al.’s study [65]. Prevention interventions conducted directly by schools prove more effective as they combine theoretical learning with the practical application of knowledge about oral hygiene [66].

The data collected in this study reflect the national situation in which most children in both countries do not have the opportunity to brush their teeth at school, with a greater number of Spanish children returning from afternoon sessions with lunch at the school cafeteria compared to Italy. Prevention projects and programs in the school environment can not only inform children about the importance of oral hygiene but also involve parents in this process. During the developmental period, habits and experiences related to food and oral hygiene become integral parts of the child’s well-being and knowledge, influencing their future quality of life.

In particular, Calcagnile et al. [63] suggest organizing primary prevention health promotion programs as follows: during pregnancy, maintain a healthy diet and good oral hygiene to reduce the cariogenic bacteria concentration before the eruption of the child’s teeth, thus preventing early transmission. From an early age, it is crucial to introduce the child to dental visits to intercept and prevent potential future oral pathologies. Additionally, introducing oral health education programs in schools is essential, even though parents with lower socioeconomic status have more difficulty accessing oral health information [67,68].

Even though the percentage of children with caries has decreased below 50% (the WHO’s goal for the year 2000) [69], the prevalence of dental caries among children in Italy and Spain remains too high. Despite easy access to information through social networks and healthcare professionals, dental caries prevalence among children is still significantly high in these examined countries. From the questionnaire data, it is evident that most parents from both nationalities use age-appropriate toothpaste for their children. However, only a fraction of them are aware of the positive effects of fluoridated products in preventing carious lesions. Unfortunately, knowledge gaps still persist, particularly among individuals with lower education levels [15].

Educating individuals about proper oral hygiene and making dental care accessible to parents and children are fundamental to support them and minimize risky behaviors that may impact their children. Pranno et al. [15] reported that mothers show more attention to their children’s oral health than fathers. Additionally, a higher level of oral health is observed in parents with a medium/high sociocultural level, as also reported in Chen et al.’s study on a sample of 8446 families [70]. Since age significantly influences concept acquisition, disseminating the importance of prevention from a young age makes the child aware of their oral health and its significance throughout life.

## 5. Conclusions

According to the November 2013 Italian ministerial guidelines [8], an individual’s risk of developing caries needs evaluation based on their caries experience, dietary habits, oral hygiene, fluoride usage, overall health, and family’s socioeconomic status. These factors are derived from assessing the importance parents place on oral health, the health education they impart to their children, and their attitude towards their children’s oral hygiene at home. Based on the results of our study, timely interventions are necessary. There is still insufficient knowledge about the origin, causes, and transmission of dental caries. It is crucial to raise awareness, especially among mothers, to prevent potential bacterial transmission from an early age, through preventive campaigns aimed at increasing their awareness of the disease and through screening and oral hygiene education starting from pregnancy.

The concept of oral disease prevention disseminated by dentists and dental hygienists involves both direct monitoring by professionals and, more importantly, managing oral hygiene habits by parents. Devoting time to instructing parents represents a comprehensive preventive methodology since they serve as behavioral role models for their children. Adopting and learning good behavioral habits during childhood begins at home and continues in both the domestic environment and school. Hence, dentists and dental hygienists play a crucial role in providing access to proper information and correct behavioral habits in oral hygiene. It is crucial to encourage both children and parents to use fluoride toothpastes with appropriate fluoride content, as recommended by International and European Pediatric Dentistry Guidelines [71,72], strengthen tooth brushing in school prevention programs, and enhance oral health care programs and educational campaigns, including the promotion of sealing pits and fissures.

## Figures and Tables

**Table 1:** Structure of the administered questionnaire.

Sections	Questions
Section I	1. Are you the mother or father? 2. Is this your first experience as a parent? 3. Gender of the child? 4. Age of the child? 5. Does your child suffer from any condition?
Section II	1. Is dental caries a disease? 2. Can dental caries be transmitted? 3. Has your child ever experienced dental caries? 4. If you answered “Yes” to the previous question, was the dental caries treated? 5. In your opinion, should decayed baby teeth be treated?
Section III	1. Does every family member have their own toothbrush? 2. Do you share the same utensils (forks and spoons) with your child during meals? 3. How was your child breastfed? (natural/artificial/mixed) 4. How long did the breastfeeding period last? 5. Has your child used or currently uses any of the following devices? (pacifier/pacifier with honey or sugar/milk in the baby bottle/none of the above) 6. If you checked any of the previous answers in the preceding question, for what reason? 7. If you checked any of the answers in the preceding question, how long has this habit been in practice?
Section IV	1. Have you ever had a dental visit before or during pregnancy? (for mothers only) 2. If you answered “Yes” to the previous question, why? 3. Did your gynecologist recommend a dental visit among the routine tests in the first trimester of pregnancy?
Section V	1. When did your child start brushing their teeth? 2. What toothpaste does your child use? 3. What do you think is the most suitable toothbrush for your child? 4. In your opinion, is tongue hygiene important?
Section VI	1. What does your child usually eat during the school break? 2. Does your child have afternoon school sessions? 3. If you answered “Yes” to the previous question, does the school offer the opportunity for children to brush their teeth?
Section VII	1. Have you ever heard of “Pit and fissure sealants”? 2. Did you administer fluoride (drops or tablets) to your child during their first 3 years of life?

**Table 2:** Children’s ages declared by parents involved in the study.

Age	Italian (%)	Spanish (%)
0–3	8.6	42.4
3–6	10.3	38.1
6–9	25.8	9.2
9–12	30.2	5.4
>12	25.1	4.9

**Table 3:** Illness or conditions present in the children in the study.

	Italian		Spanish	
Does your child suffer from any condition?	**Yes (%)**	**No (%)**	**Yes (%)**	**No (%)**
	17.5	82.5	4.6	Di

**Table 4:** Main questions in section II of the questionnaire in both groups.

	Italian			Spanish		
	Yes (%)	No (%)	I Do Not Know (%)	Yes (%)	No (%)	I Do Not Know (%)
Is dental caries a disease?	45.4	35.4	19.2	79.9	9.2	10.9
Can dental caries be transmitted?	12.4	75.6	12	25.5	65	9.5
Has your child ever experienced dental caries?	46.7	53.3	-	13.8	86.2	-
In your opinion, should decayed baby teeth be treated?	81.1	10	8.9	95.1	0.6	4.3

**Table 5:** Parental behavioral patterns.

	Italian		Spanish	
	Yes (%)	No (%)	Yes (%)	No (%)
Does every family member have their own toothbrush?	99.7	0.3	100	-
Do you share the same utensils (forks and spoons) with your child during meals?	9.6	90.4	25.8	74.2
Have you ever had a dental visit before or during pregnancy? (for mothers only)	64.6	35.4	86	14
Have you ever heard of “Pit and fissure sealants”?	40.5	59.5	37.5	62.5
Did you administer fluoride (drops or tablets) to your child in their first 3 years of life?	29.6	70.4	11.7	88.3

**Table 6:** The school’s role in oral hygiene education.

	Italian		Spanish	
	Yes (%)	No (%)	Yes (%)	No (%)
Does your child have afternoon school sessions?	39.5	60.5	66.2	33.8
If you answered “Yes” to the previous question, does the school offer the opportunity for children to brush their teeth?	24.3	75.7	29.9	70.1

## Data Availability

Data are available from the corresponding author upon request.
